# Association between maternal sleep duration and quality, and the risk of preterm birth: a systematic review and meta-analysis of observational studies

**DOI:** 10.1186/s12884-020-2814-5

**Published:** 2020-02-24

**Authors:** Ling Wang, Feng Jin

**Affiliations:** Department of Obstetrics and Gynecology, Shengjing Hospital of China Medical University, No. 36, San Hao Street, Shenyang, Liaoning 110004 People’s Republic of China

**Keywords:** Meta-analysis, Observational studies, Preterm birth, Sleep, Systematic review

## Abstract

**Background:**

To assess the association of sleep duration and quality with the risk of preterm birth.

**Methods:**

Relevant studies were retrieved from the PubMed and Web of Science databases up to September 30, 2018. The reference lists of the retrieved articles were reviewed. Random effects models were applied to estimate summarized relative risks (RRs) and 95% confidence intervals (CIs).

**Results:**

Ten identified studies (nine cohort studies and one case-controlled study) examined the associations of sleep duration and quality with the risk of preterm birth. As compared with women with the longest sleep duration, the summary RR was 1.23 (95% CI = 1.01–1.50) for women with the shortest sleep duration, with moderate between-study heterogeneity (*I*^2^ = 57.4%). Additionally, as compared with women with good sleep quality, the summary RR was 1.54 (95% CI = 1.18–2.01) for women with poor sleep quality (Pittsburgh Sleep Quality Index > 5), with high between-study heterogeneity (*I*^2^ = 76.7%). Funnel plots as well as the Egger’s and Begg’s tests revealed no evidence of publication bias.

**Conclusions:**

This systematic review and meta-analysis revealed that short sleep duration and poor sleep quality may be associated with an increased risk of preterm birth. Further subgroup analyses are warranted to test the robustness of these findings as well as to identify potential sources of heterogeneity.

## Background

Each year, more than 10% of births worldwide are preterm, occurring before gestational week 37. Prematurity, which is a leading cause of mortality among children aged less than 5 years [1, 2], leads to short and long-term morbidities, as well as serious health problems, including both physical and mental disabilities [3–5]. Recently, increasing numbers of studies have sought to identify risk factors associated with preterm birth. Moreover, a recent review summarized the risk factors for prematurity, which included pregnancy-related depression, stress, and anxiety [6], while preexisting diabetes, hypertension, sickle cell anemia, and previous preterm birth were associated with a two-fold greater risk of preterm birth [7].

Due to psychophysiological changes caused by pregnancy, approximately 27.9% of women sleep for less than 7 h per night because of sleep disturbances [8]. Thus, numerous studies have investigated the relationship between maternal sleep practices and fetal outcomes. For instance, insomnia and sleep apnea in pregnancy increased the risk of preterm birth by 30 and 40%, respectively [9]. Moreover, pregnancies complicated by restless legs syndrome are also at an increased risk for preterm birth [10], while pregnancy-related endocrinological and physical changes can also result in sleep disturbances [11]. As compared with the general population, pregnant women are more likely to encounter sleep-related disorders, including poor subjective sleep quality and shorter hours sleeping. However, a previous study reported that sleep had no association with prematurity [12], whereas others have found that a short duration and poor quality of sleep were both risk factors for preterm birth [13, 14].

A previous meta-analysis evaluated the association between sleep quality and duration with the risk of preterm birth [15], but there was significantly high heterogeneity among the included studies and no subgroup or sensitivity analysis to identify the sources of heterogeneity and test the robustness of the main findings. Since an increasing number of studies with inconsistent results have reported that preterm birth is associated with maternal sleep duration and quality, the aim of the present meta-analysis was to systematically further review the current literature regarding the impact of maternal sleep duration and quality on the risk of preterm birth.

## Material and methods

### Search strategy

After formulating the study questions, a systematic review and meta-analysis were performed in accordance with the Preferred Reporting Items for Systematic Reviews and Meta-Analyses guidelines [16]. Based on Medical Subject Heading (MeSH) terms and key words used in recent reviews [15, 17], studies were retrieved from the PubMed and Web of Science databases with restrictions to those published in English up to September 30, 2018. The complete database search strategy is described in Table 1. Additional publications were obtained by manually searching the reference sections of primary studies and review articles.

**Table 1:** The search strategy of the association of sleep duration and quality with preterm birth

#1	Search “Sleep”[MeSH]
#2	sleep duration OR sleep time OR sleep disorders OR sleep quality OR sleep deprivation OR sleep disturbance OR 24-h sleep duration OR night time sleep duration OR short sleep duration OR long sleep duration OR sleep insufficient OR sleep loss OR sleep poor OR sleep inadequate OR sleep amount OR sleep restrict OR sleep lack OR sleep impair OR hypersomnia OR daytime sleepiness OR long sleepers OR short sleepers OR sleep initiation and maintenance disorders OR nap OR napping OR siesta OR drowse OR insomnia OR drowsiness OR sleep wake disorders OR sleep stages
#3	#1 OR #2
#4	Search “Pregnancy”[MeSH]
#5	gestation OR gestational OR pregnant women OR gravidity OR pregnancies
#6	#4 OR #5
#7	Search “Premature Birth”[MeSH]
#8	infant, newborn OR infant, premature OR obstetric labor, premature OR birth, premature OR births, premature OR premature births OR birth, preterm OR births, preterm OR preterm births OR labor, premature OR infant, extremely premature OR pregnancy outcome OR birth outcomes OR labor, premature obstetric OR premature labor OR preterm labor OR labor, preterm OR labor, premature OR premature obstetric labor
#9	#7 OR #8
#10	#3 AND #6 AND #9
	Restriction to articles published in English and up to September 30, 2018.

Based on MeSH terms and key words, a strategy was created to search related literature in the PubMed and Web of Science databases

### Study selection

The titles and abstracts of the retrieved articles were screened by two independent reviewers (LW and FJ) and any disagreements were resolved by discussion. Articles that fulfilled the following criteria were eligible for the present meta-analysis: (i) participants were pregnant women who were recruited from all populations; (ii) sleep duration and quality were quantified during the term of pregnancy; (iii) preterm birth, as defined as childbirth before gestational week 37, was the primary or secondary outcome; (iv) the study used an observational design (including case-control, nested case-control, cross-sectional, and cohort studies); and (v) the relative risk (RR), hazard ratio (HR), or odds ratio (OR), and corresponding 95% confidence interval (95% CI) or necessary data for calculation were reported. Review articles, case reports, commentaries, and conference abstracts were excluded. If data were duplicated in more than one study, the study with the largest number of cases was included.

### Data extraction and quality assessment

The data from each of the included studies were extracted carefully by two independent reviewers (LW and FJ). The following information from each article was recorded in a standardized, pre-designed spreadsheet: the first author’s name; publication year; country of origin; study design; number of cases/participants; source of patient recruitment; sleep assessment; pregnancy outcome categories; exposure categories (period of exposure measurement); risk estimates from the most fully adjusted model with associated 95% CI values; and confounders adjusted for multivariate analysis.

Considering that all of the included articles were observational studies, two independent reviewers (LW and FJ) used the Newcastle–Ottawa quality assessment scale for observational studies to assess the risk of bias [18]. Subsequently, the studies that achieved a full rating in at least two categories of selection, comparability, or outcome assessment were considered at a low risk of bias [19, 20].

### Statistical analysis

For studies that separately reported results for different trimesters, but not in combination, inverse variance-weighted fixed effects meta-analysis was first used to generate a risk estimate of the overall study at full gestation before random-effects meta-analysis. For studies that failed to use the category with the shortest sleep duration as the reference, the effective count method proposed by Hamling et al. [21] was used to recalculate the risk estimates. The summary RR values and corresponding 95% CI values of the included studies were used as measures to assess the association of sleep duration and quality with the risk of preterm birth.

Between-study heterogeneity of each meta-analysis was estimated using the *I*^2^ statistic. *I*^2^ values of 25, 50, and 75% were considered to represent low, moderate, and high heterogeneity, respectively [22]. Summary RR values were calculated using the DerSimonian and Laird random-effects model. The sequential exclusion strategy proposed by Patsopoulos et al. [23] was used to determine whether the overall estimates were influenced by substantial heterogeneity. Studies that accounted for the largest share of heterogeneity were sequentially and cumulatively excluded until *I*^2^ was < 50%. The RR estimates were then assessed for consistency [23]. Potential publication bias was examined with a funnel plot and the Egger’s and Begg’s tests (*p* < 0.10). The G*Power 3.1.9.2 statistical power analysis program was used to calculate the sample size at an alpha value of 0.05, power of 80% (β = 0.2), estimated RR of 1.2, and proportion of outcome in the exposure group of 0.3. Accordingly, the lowest sample size for a proper cohort was *n* = 1138 for dichotomous outcomes. All data analyses were performed using STATA software, version 12.0 (Stata Corp, College Station, TX, USA). A probability (*p*) value of < 0.05 was considered statistically significant unless otherwise specified.

## Results

### Characteristics of the retrieved studies

**Fig. 1:**
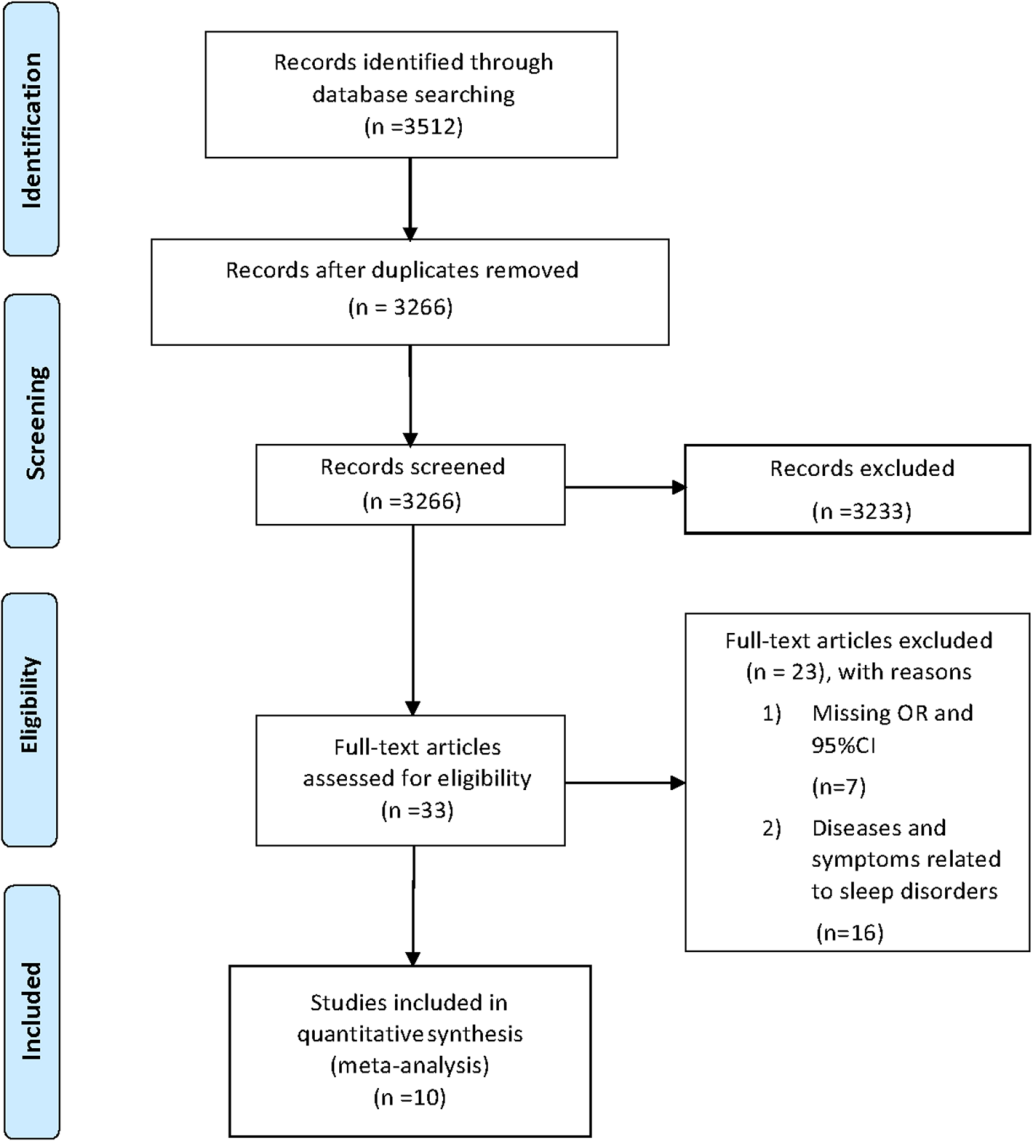
Flow chart for screening of relevant literature. Selection of studies for inclusion in the present meta-analysis

Of the 3266 retrieved studies remaining after the removal of duplicates, 3233 (99.0%) were excluded after screening of the titles and abstracts. After full text review of the remaining 33 (1.01%) studies, 16 analyzed diseases and symptoms related to sleep disorders, and seven lacked OR and 95% CI values. Among these seven studies, three suggested no association between sleep and risk of preterm birth [24–26], two indicated that short sleep was correlated with prematurity [10, 27], one showed that short and long sleep durations were associated with increased risk of preterm birth [28], and one analyzed the relationships among sleep, inflammatory cytokine levels, and preterm birth, but failed to show a direct association between sleep and preterm birth [29]. Finally, 10 published studies detailing the associations of sleep duration and quality with the risk of preterm birth were included for analysis (Fig. 1). Among these, seven and five published studies focused on sleep duration and quality, respectively.

The characteristics of the 10 studies (nine cohort studies and one case-control study) included for analysis are summarized in Tables 2 and 3. Notably, each study was published between 2011 and 2018. The number of cases in each study ranged from 12 to 479 and the number of participants/controls ranged from 116 to 1977. All of the included studies measured sleep quality and sleep duration with the use of questionnaires. The majority of studies were conducted in the USA (*n* = 5), India (*n* = 1), Greece (*n* = 1), Japan (*n* = 1), and China (*n* = 1), and were adjusted for potentially important confounders, such as body mass index (*n* = 4) and maternal age (*n* = 5). On the basis of the Newcastle–Ottawa quality assessment scale, all studies were judged to have a low risk of bias (Table 4).

**Table 2:** Characteristics of studies included in the meta-analysis

First Author, (Ref), Year, Country	Study Design	No. of Case/Study size	Source of patient recruitment	Sleep assessment	Pregnancy outcome categories	Exposure categories (period of exposure measurement)	Risk Estimates (95% CI)
Sleep Duration							
Trivedi et al., [30] 2018, India	Cohort study	180/1977	At household level	Questionnaire	Preterm birth	**Sleep hours per day** **(> 8 h versus ≤ 8 h)** during antenatal period	0.93 (0.91–0.94)
Li et al., [31] 2017, China	Cohort study	32/688	At three hospitals	Questionnaire	Preterm birth	**Average night’s sleep** **(< 7 h versus ≥ 7 h)** 12–16 weeks 24–28 weeks 32–36 weeks	0.58 (0.67–5.05) 1.56 (0.29–8.29) 4.67 (1.24–17.5)
Kajeepeta et al., [32] 2014, Peruvian	Case-control study	479/480	At hospital level	Questionnaire		**Hours of sleep per night** **0–24 weeks** **(≤6 h, ≥9 h versus 7–8 h)**	
					Spontaneous Preterm Birth	≤6 h ≥9 h	1.53 (1.08–2.17) 1.50 (1.04–2.16)
					Spontaneous Very Preterm < 32 weeks	≤6 h ≥9 h	1.25 (0.74–2.11) 1.48 (0.89–2.47)
					Spontaneous Moderate Preterm 32–33 weeks	≤6 h ≥9 h	1.82 (0.98–3.37) 1.98 (1.08–3.65)
					Spontaneous Late Preterm 34- < 37 weeks	≤6 h ≥9 h	1.64 (1.11–2.43) 1.10 (0.72–1.70)
					Spontaneous Preterm Labor	≤6 h ≥9 h	1.51 (1.00–2.29) 1.50 (0.99–2.27)
					Preterm Premature Rupture of Membrane	≤6 h ≥9 h	1.60 (1.06–2.40) 1.17 (0.74–1.83)
Guendelman et al., [12] 2013, USA	Cohort study	344/698	A cohort	Questionnaire	Preterm birth	**Total sleep duration** **12–24 weeks** **(< 7 h, > 8 h versus 7-8 h)** < 7 h > 8 h	1.09 (0.80–1.48) 0.88 (0.57–1.48)
Okun et al., [33] 2012, USA	Cohort study	26/217	Self-referral, physician referral, advertising, and screening in obstetrical ultrasound suites	Questionnaire	Preterm birth	**Total sleep duration** **(< 7 h versus ≥ 7 h)** at 20 week at 30 week **(> 9 h versus ≤ 9 h)** at 20 week at 30 week	0.86 (0.29–2.59) 0.97 (0.31–3.02) 1.19 (0.38–3.75) 0.88 (0.20–3.91)
Reutrakul et al., [34] 2011, USA	Cohort study	56/116	In all population	Questionnaire	Preterm birth	**Sleep duration per night** **(< 7 h versus ≥ 7 h)** Second trimester	4.3 (1.1–16.7)
Micheli et al., [13] 2011, Greece	Cohort study	131/1091	The Mother-child Cohort	Questionnaire		**Sleep duration** **24–36 weeks** **(≤5 h, 6-7 h versus ≥ 8 h)**	
					All preterm	≤5 h 6-7 h	1.7 (1.1–2.8) 0.9 (0.6–1.4)
					Medically Indicated Preterm	≤5 h 6-7 h	2.4 (1.0–6.4) 1.2 (0.5–2.7)
					Spontaneous Preterm	≤5 h 6-7 h	1.6 (0.8–2.9) 0.9 (0.6–1.4)
Sleep Quality							
Li et al., [31] 2017, China	Cohort study	32/688	At three hospitals	Questionnaire	Preterm birth	**(PSQI > 5 versus PSQI≦5)** 12–16 weeks 24–28 weeks 32–36 weeks	2.04 (0.65–6.43) 5.35 (2.10–13.63) 3.01 (1.26–7.19)
Ota et al., [35] 2017, Japan	Cohort study	23/122	At hospital	Questionnaire	Preterm birth	**(PSQI > 5 versus PSQI≦5)** at 16 week	3.4 (1.2–9.9)
Blair et al., [36] 2015, USA	Cohort study	12/138	The Ohio State University Wexner Medical Center and the surrounding community of Columbus	Questionnaire	Preterm birth	**(PSQI > 5 versus PSQI≦5)** Mid-pregnancy	4.11 (1.04–16.25)
Reutrakul et al., [34] 2011, USA	Cohort study	74/116	In all population	Questionnaire	Preterm birth	**(PSQI > 5 versus PSQI≦5)** Second trimester	1.2 (1.0–1.3)
Okun et al., [14] 2011, USA	Cohort study	15/166	A large university medical center	Questionnaire	Preterm birth	**(PSQI > 5 versus PSQI≦5)** 14–16 weeks 30–32 weeks	1.25 (1.04–1.50) 1.18 (0.98–1.42)

The characteristics of the 10 studies (nine cohort studies and one case-controlled study) included for analysis are summarized in this table. The basic extracted information included the first author’s name; publication year; country of origin; study design; number of cases and populations; source of patient recruitment; sleep assessment; pregnancy outcome categories; exposure categories (period of exposure measurement); and risk estimates from the most fully adjusted model with associated 95% CI values

*Ref*, Reference, *CI* Confidence interval, *H* Hours, *NA* Not available, *PSQI* Pittsburgh Sleep Quality Index

**Table 3:** Matched or adjustment of potential confounders of studies included in the meta-analysis

First Author, (Ref), Year, Country	Matched or adjusted factors
Sleep Duration	
Trivedi et al., [30] 2018, India	Age at pregnancy, caste, religion, occupation, literacy, and number of Ante Natal Care visits
Li et al., [31] 2017, China	Pre-pregnancy body mass index and birth weight
Kajeepeta et al., [32] 2014, Peruvian	Maternal age, pre-pregnancy weight, unplanned pregnancy, and no vitamin use during pregnancy
Guendelman et al., [12] 2013, USA	Race and month of delivery
Okun et al., [33] 2012, USA	Major depressive disorder, selective serotonin reuptake inhibitors, age, employment, marital status, history of preterm birth
Reutrakul et al., [34] 2011, USA	Body mass index
Micheli et al., [13] 2011, Greece	Maternal age, education, parity, smoking during pregnancy, pre-pregnancy body mass index
Sleep Quality	
Li et al., [31] 2017, China	Pre-pregnancy body mass index and birth weight
Ota et al., [35] 2017, Japan	Infertility treatment, asthma, and history of alcohol use
Blair et al., [36] 2015, USA	Age
Reutrakul et al., [34] 2011, USA	Body mass index
Okun et al., [14] 2011, USA	Income and medical risk factors

The matched or adjustment of potential confounders of the studies included in the meta-analysis are summarized in this table

**Table 4:** Methodological quality of observational studies included in the meta-analysis

First author, (Ref), year	Selection				Comparability	Outcome		
Cohort study	Representativeness of the exposed cohort	Selection of the non-exposed cohort	Ascertainment of exposure	Demonstration that outcome of interest was not present at start of study	Comparability of cohorts on the basis of the design or analysis	Assessment of outcome	Was follow-up long enough for outcomes to occur	Adequacy of follow up of cohorts
Trivedi et al., [30] 2018	☑	☑	☑	☑	–	☑	☑	☑
Li et al., [31] 2017	☑	☑	☑	☑	☑	☑	☑	☑
Ota et al., [35] 2017	☑	☑	☑	☑	☑	☑	☑	☑
Blair et al., [36] 2015	☑	☑	☑	☑	☑	☑	☑	☑
Guendelman et al., [12] 2013	☑	☑	☑	☑	☑	☑	☑	☑
Okun et al., [33] 2012	☑	☑	☑	☑	☑☑	☑	☑	☑
Reutrakul et al., [34] 2011	☑	☑	☑	☑	☑	☑	☑	☑
Okun et al., [14] 2011	☑	☑	☑	☑	☑	☑	☑	☑
Micheli et al., [13] 2011	☑	☑	☑	☑	☑☑	☑	☑	☑
**First author, (Ref), year**	**Selection**				**Comparability**	**Exposure**		
Case-control study	Is the case definition adequate	Representativeness of the cases	Selection of Controls	Definition of Controls	Comparability of cases and controls on the basis of the design or analysis	Ascertainment of exposure	Same method of ascertainment for cases and controls	Non-Response rate
Kajeepeta et al., [32] 2014	☑	☑	☑	☑	☑☑	☑	☑	☑

The risk of bias of all studies included in this meta-analysis was based on the Newcastle–Ottawa quality assessment scale

The definition/explanation of each column of the Newcastle–Ottawa Scale is available at http://www.ohri.ca/programs/clinical_epidemiology/oxford.asp

### Sleep duration and risk of preterm birth

**Fig. 2:**
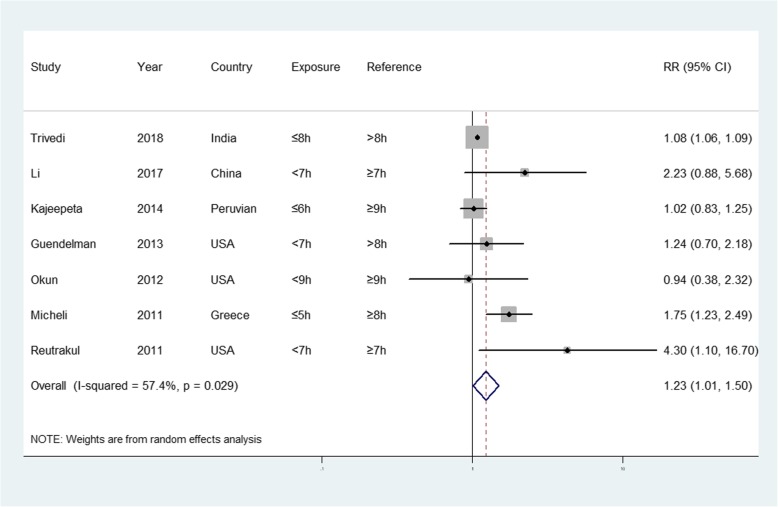
Forest plot (random-effects model) of sleep duration (shortest vs. longest) and preterm birth risk. The squares indicate study-specific hazard ratios (size of the square reflects the study-specific statistical weight); the horizontal lines indicate 95% CIs; and the diamond indicates the summary hazard ratio estimate with the related 95% CIs. CI, confidence interval; RR, relative risk

**Fig. 3:**
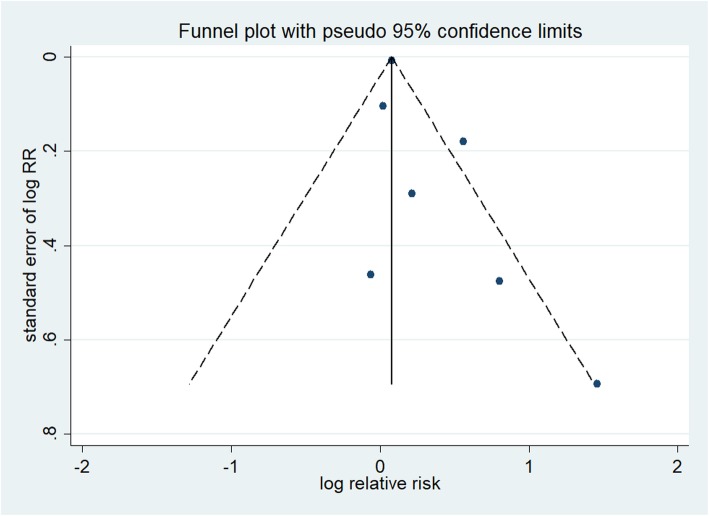
Funnel plot corresponding to the random-effects meta-analysis of the relationship between sleep duration and preterm birth risk

The forest plots in Fig. 2 show the summarized results for the association between sleep duration and the risk of preterm birth risk in seven of the included studies, involving 1248 preterm birth cases and 5267 participants [12, 13, 30–34]. Women with the shortest sleep duration were 1.23 times more likely to have a preterm birth than those with the longest sleep duration (summarized RR = 1.23; 95% CI = 1.01–1.50). Moderate heterogeneity was observed (*I*^2^ = 57.4%, *p* = 0.029). Funnel plots as well as the Egger’s and Begg’s tests (*p* = 0.123 and 0.133, respectively) revealed no evidence of publication bias (Fig. 3).

The summarized RR values ranged from 1.41 (95% CI = 1.01–1.96, *I*^2^ = 63.7%; exclusion of Kajeepeta et al. [32]) to 1.40 (95% CI = 0.99–1.99, *I*^2^ = 58.9%; exclusion of Trivedi et al. [30]). After excluding studies that failed to adjust for potential confounders, the results were robust (RR = 1.31, 95% CI = 0.95–1.79) without heterogeneity (*I*^2^ = 54.5%).

### Sleep quality and preterm birth risk

**Fig. 4:**
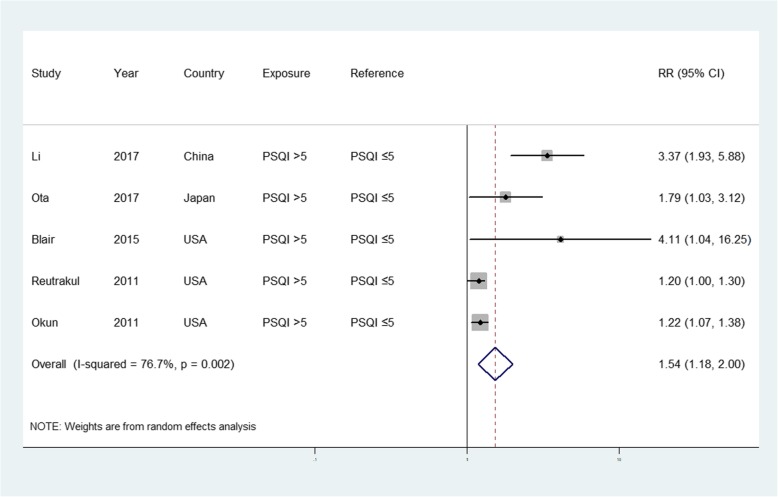
Forest plot (random-effects model) of sleep quality (poor vs. good) and preterm birth risk. The squares indicate study-specific hazard ratios (size of the square reflects the study-specific statistical weight); the horizontal lines indicate 95% CIs; and the diamond indicates the summary hazard ratio estimate with the related 95% CIs. CI, confidence interval; RR, relative risk

**Fig. 5:**
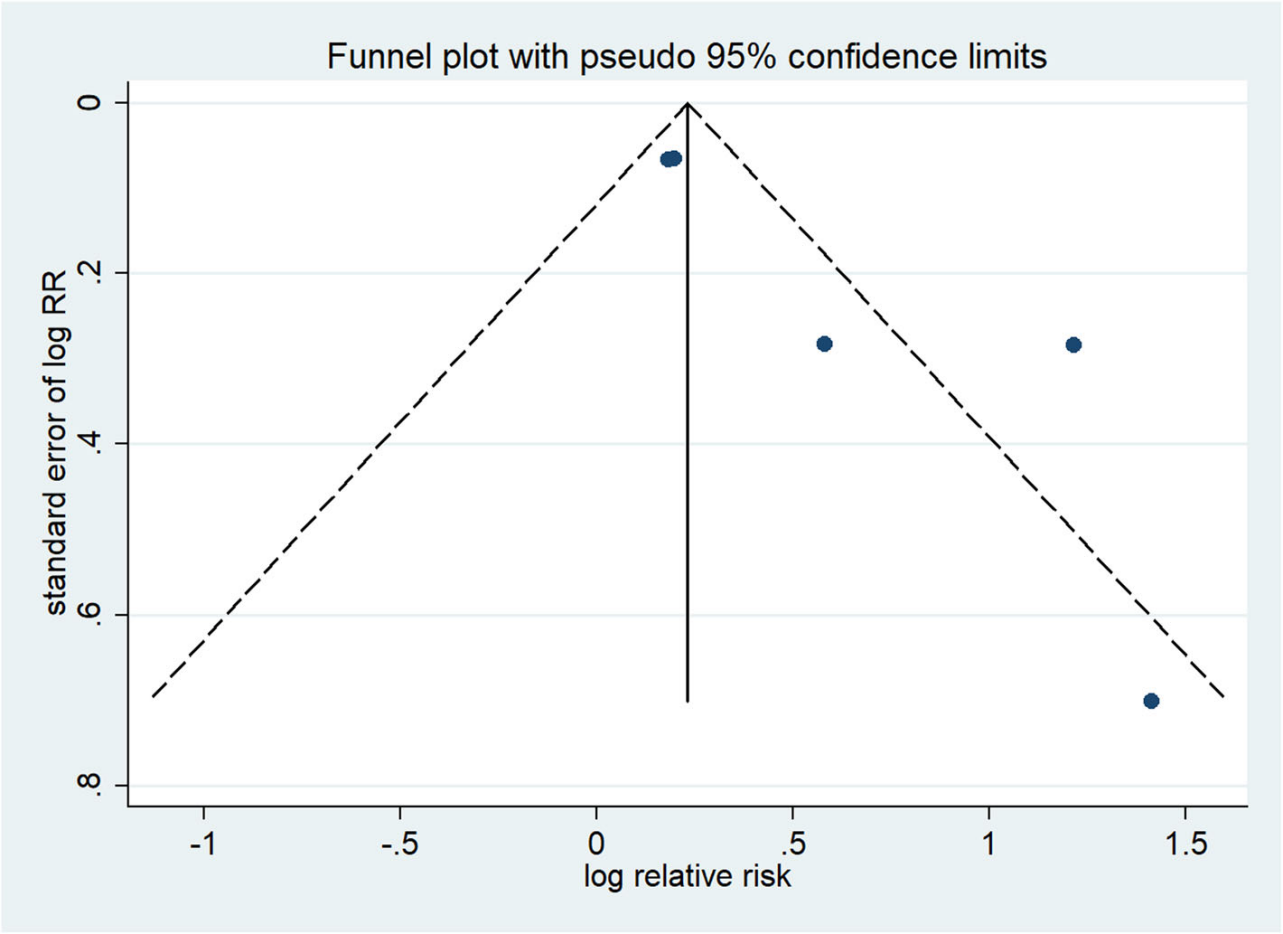
Funnel plot corresponding to the random-effects meta-analysis of the relationship between sleep quality and preterm birth risk

Five of the included studies with 156 preterm birth cases and 1230 participants examined the association between sleep quality and the risk of preterm birth [14, 31, 34–36]. The forest plots in Fig. 4 show the summarized results for the association between sleep quality and the risk of preterm birth. Women with poor sleep quality (Pittsburgh Sleep Quality Index [PSQI] > 5) were 1.54 times more likely to have a preterm birth than those with good sleep quality (summarized RR = 1.54; 95% CI = 1.18–2.01). High heterogeneity was observed (*I*^2^ = 76.7%, *p* < 0.001). Funnel plots as well as the Egger’s and Begg’s tests (*p* = 0.221 and 0.139, respectively) revealed no evidence of publication bias (Fig. 5).

When the studies contributing to the largest extent of heterogeneity were sequentially excluded until *I*^2^ was < 50% [31], the summarized RR values for preterm birth (RR = 1.26, 95% CI = 1.09–1.45, *I*^2^ = 38.4%) were similar to the original estimates. Additionally, the summarized RR values ranged from 1.26 (95% CI = 1.09–1.45, *I*^2^ = 38.4%; exclusion of Li et al. [31]) to 2.05 (95% CI = 1.12–3.75, *I*^2^ = 82%; exclusion of Okun et al. [33]).

## Discussion

The present systematic review and meta-analysis of 10 observational studies demonstrated that short sleep duration and poor sleep quality were associated with a significantly increased risk of preterm birth. This association highlights the vital significance of pregnant women to reduce the risk of premature birth.

In regard to sleep duration and quality, the inconsistent findings of previous studies might be attributed to differences in the trimester examined and geographical location. For instance, Micheli et al. [13] conducted a cohort study (*n* = 1091), in which 23% of pregnant women reported a sleep duration of ≤5 h in the third trimester. However, Reutrakul et al. [34] reported that about 56% of pregnant women experienced sleep deprivation in the second trimester, but this study had a relatively small cohort (*n* = 116). Meanwhile, Li et al. [31] enrolled participants with a similar proportion of short sleepers in all trimesters and the results were similar with the main findings. One study [34] reported that about 50% of pregnant women experienced sleep deprivation, but the sleep durations differed (< 7 and < 8 h/night, respectively). Furthermore, preterm birth rates vary among countries, even different regions in the same country. Also, income and education differences may affect sleep duration and preterm birth rates [37, 38]. Warland et al. [15] speculated that African Americans may exhibit heightened sensitivity to the adverse physiological sequelae of poor sleep quality. Two other studies indicated that pregnant women with clinically disturbed sleep (PSQI > 5) accounted for a similar proportion (about 60%), despite the study being conducted in various regions within the USA [34, 36], while a study conducted in Japan included a lower proportion of poor sleepers at the initial examination and gestational weeks 16, 24, and 32 (27, 34, 37, and 41% of the samples, respectively) [35]. In addition, a study conducted in the USA reported that pregnant women at gestational week 14–16 accounted for 36.4% (*n* = 48) of the sample [14].

The findings of the current study raise questions about the potential mechanisms underlying the increased risk of preterm birth due to sleep disorders. Sleep deprivation partially accounts for the proinflammatory cytokine response [39–41], immune changes [42], and greater susceptibility to infections [43]. It is well established that inflammation and infection are highly significant risk factors for preterm birth [44, 45]. Additionally, a short sleep duration and poor sleep quality may result from stress and as a physiological stressor per se, stress “overload” and activation of the stress system may lead to prematurity through impairment of the hypothalamus-pituitary-adrenal axis and activation of the proinflammatory system [46]. On the other hand, physiological and hormonal changes also affect sleep practices. For instance, higher levels of estrogen and progesterone during pregnancy contribute to poor sleep quality and also influence the secretion of other hormones, such as cortisol and melatonin, which can increase arousal [47, 48]. Lastly, because disturbed sleep may disrupt normal remodeling of the maternal blood vessels and increased sympathetic activity, placental blood flow was decreased [49, 50], which may be a mechanism underlying preterm birth.

The strengths of the present meta-analysis lie in its quantitative analysis of the association of sleep duration and quality with the risk of prematurity using a large number of participants (*n* = 5693) and instances of preterm birth associated with sleep duration (*n* = 1248) and sleep quality (*n* = 156). The large sample size of this meta-analysis provide strong power for the main analyses and the conclusions derived. Furthermore, numerous sensitivity analyses showed that the main findings were robust. Of note, quality assessment showed that all of the included studies were at a low risk of bias.

Findings from the present meta-analysis should be interpreted in light of several limitations. First, the present meta-analysis was prone to inherent recall and selection bias due to the inclusion of original observational studies. Although case-control studies are more susceptible to bias than cohort studies, the results were robust after exclusion of the only case-control study from the analyses. Furthermore, the PSQI is an important clinical and research tool to gauge sleep quality [51]. However, the PSQI includes sleep duration, thus short sleep duration was included as an outcome of “sleep quality.” Consistently, the pooled effect size for poor sleep quality (RR = 1.54) was similar to that for a shorter duration (RR = 1.32), as determined by the meta-analysis. Moreover, in consideration of the variation of the study populations, geographical location likely contributed to the heterogeneity of effect estimates. Furthermore, since all of the included studies measured sleep quality and sleep duration with the use of questionnaires, self-reported sleep quality and duration are not always perfectly aligned with objective sleep quality and duration. Third, because the pooled effect estimates were mostly derived from observational studies, susceptibility to confounding factors remain a concern.

Some common chronic diseases, as mediators between short sleep duration and preterm birth, such as diabetes [34, 52], hypertension [53, 54], and obesity [55], have been correlated with prematurity. Of note, self-reported sleep disturbances are predictive of the incidence of major depression and strongly precede a series of symptoms of depression [56, 57]. The association between depression syndrome and the risk of preterm delivery has been reported [58]. Thus, early intervention to prevent poor sleep quality and a short sleep duration, which may be indicators of early depression, can reduce the risk of preterm birth. However, the observational studies included in this meta-analysis were restricted by the lack of controls for these potentially relevant confounders. Hence, further studies are warranted with better designs to take these confounders or mediators into account or fully adjust for these confounders in order to better rule out the potential effects of residual confounding. Fourth, as the comparison of sleep duration differed considerably among the included studies, dose–response analysis was not conducted. Notably, several of the included studies suggested a potential U-shaped association between sleep duration and preterm birth. Additionally, one of the included studies suggested a potential non-linear (U-shaped) association between sleep duration and preterm birth [32]. However, since a limited number of the included studies met the criteria of linear/non-linear dose-response analysis, such analysis was not conducted in the present study. Also, although seven studies were excluded due to the lack of risk estimates for the association between sleep quantity/quality and preterm birth [10, 24–29], findings of three of these studies support the main findings of the present meta-analysis [10, 27, 28]. Of note, although the power of the main analysis suggested that the statistical power of this meta-analysis was greater than 80% to identify sleep duration/quality for preterm birth with minimum OR values of 1.20 (risk factor for sleep duration) and 1.5 (risk factor for sleep quality), limited sample sizes restricted subgroup analyses stratified by study characteristics and potential confounders. Therefore, on the basis of these limitations, priority should be given to large, adequately powered, cohort studies using standard definitions of maternal sleep duration and quality with effective data analyses. Furthermore, more comparison groups in the primary studies are needed to evaluate the possible non-linear aforementioned association.

## Conclusion

In summary, the results of this meta-analysis provide vital insights into the association of short sleep duration and poor sleep quality with a significantly increased risk of preterm birth. These findings may help researchers to identify women at risk of pre-term birth early during pregnancy to provide targeted interventions.

## Data Availability

All data generated or analyzed in this study are included in the referenced articles.

## Abbreviations

95% CI: 95% confidence interval
HR: Hazard ratio
NA: Not available
OR: Odds ratio
PSQI: Pittsburgh sleep quality index.
Ref: Reference
RR: Relative risk
